# Identification of HDAC10 as a candidate oncogene in clear cell renal carcinoma that facilitates tumor proliferation and metastasis

**DOI:** 10.1186/s13000-024-01493-2

**Published:** 2024-09-05

**Authors:** Luojia Yang, Qin Wei, Xinran Chen, Yang Yang, Qingbo Huang, Baojun Wang, Xin Ma

**Affiliations:** 1 Medical School of Chinese PLA, Beijing, 100853 China; 2 Department of Urology, The Third Medical Center, Chinese PLA General Hospital, Beijing, 100853 China; 3 The Second Hospital, Cheeloo College of Medicine, Shandong University, Jinan, 250033 China; 4 Department of Ophthalmology, Shanghai Ninth People’s Hospital, Shanghai Jiao Tong University School of Medicine, Shanghai, 200125 China; 5 Shanghai Key Laboratory of Orbital Diseases and Ocular Oncology, Shanghai, 200125 China

**Keywords:** HDAC10, Clear cell renal cell carcinoma, Deacetylation, Prognosis

## Abstract

**Background:**

Clear cell renal cell carcinoma (ccRCC) remains one of the most lethal urological malignancies even though a great number of improvements in diagnosis and management have achieved over the past few decades. Accumulated evidence revealed that histone deacetylases (HDACs) play vital role in cell proliferation, differentiation and apoptosis. Nevertheless, the biological functions of histone deacetylation modification related genes in ccRCC remains poorly understood.

**Method:**

Bulk transcriptomic data and clinical information of ccRCC patients were obtained from the TCGA database and collected from the Chinese PLA General Hospital. A total of 36 histone deacetylation genes were selected and studied in our research. Univariate cox regression analysis, least absolute shrinkage and selection operator (LASSO) regression, random forest (RF) analysis, and protein-protein interaction (PPI) network analysis were applied to identify key genes affecting the prognosis of ccRCC. The ‘oncoPredict’ algorithm was utilized for drug-sensitive analysis. Gene Set Enrichment Analysis (GSEA) and Kyoto Encyclopedia of Genes and Genomes (KEGG) enrichment analysis was used to explore the potential biological function. The ssGSEA algorithm was used for tumor immune microenvironment analysis. The expression levels of HDAC10 were validated by RT-PCR and immunohistochemistry (IHC). 5-ethynyl-2′-deoxyuridine (EdU assay), CCK-8 assay, cell transwell migration and invasion assay and colony formation assay were performed to detect the proliferation and invasion ability of ccRCC cells. A nomogram incorporating HDAC10 and clinicopathological characteristics was established to predict the prognosis of ccRCC patients.

**Result:**

Two machine learning algorithms and PPI analysis identified four histone deacetylation genes that have a significant association with the prognosis of ccRCC, with HDAC10 being the key gene among them. HDAC10 is highly expressed in ccRCC and its high expression is associated with poor prognosis for ccRCC patients. Pathway enrichment and the experiments of EdU staining, CCK-8 assay, cell transwell migration and invasion assay and colony formation assay demonstrated that HDAC10 mediated the proliferation and metastasis of ccRCC cells and involved in reshaping the tumor microenvironment (TME) of ccRCC. A clinically reliable prognostic predictive model was established by incorporating HDAC10 and other clinicopathological characteristics ( https://nomogramhdac10.shinyapps.io/HDAC10_Nomogram/ ).

**Conclusion:**

Our study found the increased expression of HDAC10 was closely associated with poor prognosis of ccRCC patients. HDAC10 showed a pro-tumorigenic effect on ccRCC and promote the proliferation and metastasis of ccRCC, which may provide new light on targeted therapy for ccRCC.

**Supplementary Information:**

The online version contains supplementary material available at 10.1186/s13000-024-01493-2.

## Introduction

Renal cell carcinoma (RCC) is a malignant tumor that arises from renal tubular epithelial cells, ranking third the most common malignant tumors in the urogenital system worldwide [1]. As the most common pathological type of RCC, clear cell renal carcinoma (ccRCC) accounts for approximately 75% of all RCC cases [2]. Nephrectomy is the primary curative modality for patients with early-stage ccRCC presently. However, due to latent symptoms in early-stage ccRCC and lack of awareness in cancer screening, patients regularly had already progressed to advanced ccRCC upon diagnosis [3]. Patients with advanced ccRCC generally have a poor prognosis as a result of the heterogeneity of ccRCC and its inherent resistance to traditional chemotherapy and radiotherapy [4]. With the advent of systemic ccRCC treatment modalities, including the application of tyrosine kinase inhibitors (TKIs), vascular endothelial growth factor receptors (VEGFRs), mammalian target rapamycin (mTOR) pathway inhibitors, and immune checkpoint inhibitors, has shown promising therapeutic efficacy [5]. Unfortunately, patients with advanced ccRCC often experience severe side effects and drug resistance [6], hence the majority of ccRCC patients do not show sustained clinical benefits. Therefore, necessitating the pursuit of forepart biomarkers, predictive models, and novel therapeutic targets.

Histone deacetylation is a widespread epigenetic modification mechanism that helps regulate chromatin structure, DNA repair, and gene expression. This process is regulated by proteins which can be categorized into three groups: “writer”, “reader”, and “eraser” [7]. Histone deacetylase enzymes (HDACs) catalyze the removal of acetyl groups from ε-N-acetyl lysine residues on both histone and non-histone proteins [8]. In humans, HDACs have been identified and are categorized into four classes based on their similarity to their yeast counterparts. HDAC10, as a class II histone deacetylase, consists of 669 amino acid residues [9]. This residue contains an N-terminal active deacetylase domain and a C-terminal leucine-rich domain (LRD) that is catalytically inactive [10]. Current studies have shown that HDAC10 can participate in the regulation of tumorigenesis and development of various tumors [11–13]. Nevertheless, the biological function of HDAC10 in ccRCC remains poorly understood.

Objective of the current study was to identify the key histone deacetylation genes affecting the prognosis of ccRCC patients and investigate its clinical significance and function. We found that both the mRNA and protein levels of HDAC10 were elevated in ccRCC, which promised a poor prognosis of ccRCC patients. In both tissue and cell lines of ccRCC, a positive correlation with high levels of HDAC10 has been observed. Such correlation was associated withpoor prognosis for ccRCC. Subsequently, knockdown of HDAC10 significantly inhibited the proliferation, migration and invasion of ccRCC cells. In summary, our findings suggest that HDAC10 have a pro-tumorigenic effect on ccRCC, providing new light on targeted therapy for ccRCC.

## Method

### Data source

The RNA-seq transcriptomic data and clinical information of 542 kidney clear cell carcinoma (KIRC) samples and 72 normal samples were obtained via The Cancer Genome Atlas (TCGA) database (https://portal.gdc.cancer.gov/). After eliminating duplicate samples or samples without clinical information, as well as those with zero survival time, a total of 525 KIRC samples and 72 normal samples were finally included in our study. In addition, three microarray datasets (accession numbers: GSE53757, GSE53000 and GSE29609) were downloaded from the GEO database. E-MTAB-1980, Miao and Kaplan-Meier Plotter cohorts were obtained from two previously published articles and Kaplan-Meier Plotter database [14–16]. A list of 36 acknowledged histone deacetylation genes was obtained from an article published by Yuyan Xu et al. [17].

### The identification of prognostic histone deacetylation genes in KIRC

Initially, the GENEMANIA database ( https://genemania.org/ ) was used to construct a protein-protein interaction (PPI) network based on the selected 36 histone deacetylation genes [18]. The Cytoscape software (Version 3.10.1) was used to visualize the network, while the cytoHubba plugin was used to screen hub genes [19]. Subsequently, the histone deacetylation genes with prognostic value were preliminarily obtained by univariate cox analysis. To avoid omissions, we adjusted the cut-off *p*-value to 0.1. Two machine learning algorithms were utilized to screen the key genes affecting the prognosis of KIRC patients. The package ‘randomForest’ was employed to build a random forest (RF) model to filter genes. The number of decision trees was set to 1000 to obtain a stable model error and high accuracy [20]. The top 15 genes with the utmost significance were screened for the subsequent analysis [20]. The ‘glmnet’ package was used to perform the least absolute shrinkage and selection operator (Lasso) analysis, which is a biased estimation for data with multiple covariances that improve statistical models’ prediction accuracy and understandability [21]. A Venn plot is used to represent the intersection set of genes obtained from the above three screening methods.

### The differential expression analysis, prognostic analysis and clinical subgroup analysis

Based on the pre-processed RNA-seq data from the TCGA-KIRC cohort, the R package ‘DESeq2’ was used to identify the differentially expressed genes (DEGs) between KIRC tissues and normal tissue with the threshold of adjusted *p*-value < 0.05 and | Log2fold change| >1 [22]. The protein expression data of HDAC10 were obtained and analyzed from the UALCAN database (http://ualcan.path.uab.edu/) [23]. A receiver operating characteristic (ROC) curve was used to evaluate the diagnostic significance of HDAC10 using the ‘plotROC’ R package. The ‘survival’ and ‘survminer’ package were utilized to perform Kaplan-Meier (KM) analysis between high and low HDAC10 groups. The clinical subgroup analysis was employed to explore the differences between high and low HDAC10 groups in vital status, T stage, N stage, M stage and pathological stage.

### **The drug sensitivity analysis, functional enrichment analysis and tumor immune infiltration analysis**

The relationship between HDAC10 and drug sensitivity was analyzed using the GSCA database (http://bioinfo.life.hust.edu.cn/GSCA/#/drug) [24]. The ‘oncoPredict’ package was used to perform the drug sensitivity analysis [25]. Gene Set Enrichment Analysis (GSEA) and Kyoto Encyclopedia of Genes and Genomes (KEGG) analysis were performed by using the ‘clusterProfiler’ R package based on the c2 (c2.all.v7.0.entrez.gmt) from the Molecular Signatures Database (MSigDB) [26–29]. The function or pathway termed with adjusted *p*-value < 0.05 and false discovery rate (FDR) < 0.25 was considered statistically significant enrichment. We utilized the single-sample gene set enrichment analysis (ssGSEA) algorithm to calculate the relative enrichment of 24 types of immune cell infiltration in KIRC tumor tissue based on gene expression profiles of each tumor sample [30]. Subsequently, we explored the differences of immune cell enrichment between the high and low HDAC10 groups and the correlation between the HDAC10 and the different immune cell infiltration.

### The establishment and evaluation of a prognostic nomogram

The clinical information (age, gender, T, N, and pathological stage), combined with HDAC10 expression level, was involved in both univariate and multivariate cox regression analysis. On the criterion of minimum AIC (Akaike information criterion), the nomogram was constructed by multivariate cox and stepwise regression analysis and presented by the ‘regplot’ R package. A user-friendly online website was created through the ‘DynNom’ package. Through the ‘rms’ and ‘ggDCA’ R packages, calibration plots and decision curve analysis (DCA) were used to evaluate the efficacy of the nomogram. The receiver operating characteristic (ROC) analysis was performed by the “riskRegression” R package [31].

### Cell lines and culture

The human ccRCC cell lines 786-O, OSRC-2, A498, SN12-PM6 and Caki-1 were purchased from the National Platform of Experimental Cell Resources for Sci-Tech (Beijing, China) and then preserved in our laboratory. 786-O and OSRC-2 cells were cultured in RPMI 1640 (Basal Media), A498 cells were cultured in minimum essential medium (MEM) (Basal Media), SN12-PM6 cells were cultured in Dulbecco’s modified Eagle medium (DMEM) (Basal Media), Caki-1 cells were cultured in McCoy’s 5 A (Basal Media), and all of which were supplemented with 10% fetal bovine serum (FBS) (Gibco) and 1% penicillin–streptomycin (Basal Media). All cells were cultured at 37 °C in a humidified incubator containing 5% CO_2_ under mycoplasma-free conditions.

### Protein extraction and Western blot

Total protein was purified from ccRCC cells using the radioimmunoprecipitation assay buffer (RIPA buffer) (Beyotime) containing protease inhibitor and phosphatase inhibitor after washing the cells twice with ice cooled PBS for 15 min. The samples were centrifuged at 12,000 rpm at 4 °C for 18 min to collect supernatants. Protein concentration was measured using the bicinchoninic acid (BCA) protein assay kit (Beyotime). After that, the protein samples (20 µg each loading) were separated in 8–12% sodium dodecyl sulfate–polyacrylamide gel electrophoresis (SDS-PAGE) gels and then transferred onto the polyvinylidene fluoride (PVDF) membranes (Millipore) in an ice bath.

For Western blot, the membranes were incubated in 5% non-fat milk solution in PBS for 2 h at room temperature and then, with primary antibodies against HDAC10 (Rat. #AF5457; Affinity; 1:1000), β-actin (Rat. #4970; Cell Signaling Technology; 1:1000) at 4 °C overnight. The membranes were incubated with secondary horseradish peroxidase conjugated antibodies goat anti-rabbit IgG (Rat. # ZB-2301; Zhongshan Golden Bridge Biotechnology; 1:5000) Blots were detected using an enhanced chemiluminescence kit (catalog E412-01, Vazyme). Protein band densities were quantified using Image-J software.

### RNA isolation and PCR analysis

According to the manufacturer’s instructions, total RNA was extracted from cultured cells or fresh tissues with a Cell Total RNA Isolation Kit (Vazyme). Total RNA was reverse transcribed into cDNAs using SureScript™ RTase Mix and SureScript™ RT Reaction Buffer for qPCR (GeneCopoeia), and qPCR was performed with 5×Blaze Taq™ qPCR Mix (GeneCopoeia) and the Applied Biosystems QuantStudio3. GAPDH served as the endogenous control. The 2^−ΔΔCT^ method was used for the relative quantification of the qPCR data. To examine alternative spliced products, BiOptic’s Qsep100 Bio-Fragment Analyzer (BiOptic) was combined with semiquantitative RT-PCR. Primer sequences were designed for the constitutively expressed flanking exons51, and 2×Taq Master Mix (Dye Plus) (Vazyme, P112-01) was used to simultaneously amplify isoforms that included or skipped the target exon. The primer for HDAC10 sequences was 5′- AGAAACACGGGCTACACAGG-3′, 5′- GCATCTGACTCTCGCAGGAA-3′.

### Immunohistochemistry (IHC)

For all human tissues and xenograft tumor tissues, IHC was performed as described previously [10]. The primary antibodies included anti-HDAC10 (Rat. #AF5457; Affinity; 1:100). The IHC scoring was performed independently by three experienced pathologists. The percentage of positive cells was classified as follows: = 0, 11–25% = 1, 26–50% = 2, 51–75% = 3, and > 75% = 4. The staining intensity was classified as follows: no staining = 0, weak intensity = 1, moderate-intensity = 2, and strong intensity = 3. The final IHC score was calculated by multiplying the staining intensity and the proportions of stained cells. Finally, tissues with scores ≥ 6 were defined as showing high expression, whereas the others were defined as showing low expression.

### 5-ethynyl-2′-deoxyuridine (EdU) staining

EdU staining was performed with EdU Imaging Kits (Cy3) (RIB BIO) according to the manufacturer’s protocol. Inoculate 2 × 10^5^ cells per well in a 24-well plate, and place cell slides in the wells. Dilute the EdU solution with complete cell culture medium at a ratio of 1000:1, add 500 µL of 50µM EdU to each well for incubation for half an hour. Wash twice with PBS for 5 minutes each time. Add 500µL of 4% paraformaldehyde to each well for incubation at room temperature for 30 min. Add 500 µL of 2 mg/mL glycine to each well, followed by the addition of 500µL of PBS per well, and wash with a decolorizing shaker for 5 minutes. Add 500µL of 1xApollo staining reaction solution to each well, incubate for 30 min in the dark at room temperature on a decolorizing shaker, and discard the staining reaction solution. Add 500µL of 0.5% TritonX-100 to each well on a decolorizing shaker for washing twice for 10 min each time, and discard the permeate. Add 100µL of 1xHoechst33342 reaction solution to each well, incubate for 30 min in the dark at room temperature on a decolorizing shaker, discard the staining reaction solution, and observe.

### Cell transwell migration and invasion assay

Cells were seeded in transwell chambers with an 8 µM pore polyethylene terephthalate filter membrane (Corning). The chamber membranes were coated with 20 µL Matrigel (Corning) before cell seeding for the invasion assay. After incubation, the chamber membranes were fixed with 4% paraformaldehyde and stained with 1% crystal violet, and the cells on the upper side of the chamber membranes were removed. The number of cells that invaded through the membrane was visually counted in three random microscopic fields (100× magnification).

### Colony formation assay

Cells were seeded into 6-well plates with a population of 1 × 10^3^ per well. After 2 weeks of culture, colonies were scored after fixing with methanol for 30 min and staining with 0.2% crystal violet.

### CCK8 assay

After digestion, counting and centrifugation, overexpression cells or knockdown cells were seeded into 96-well plates with 5000 cells per well, cultured for 24 h, and finally evaluated using the Cell Counting Kit 8 (MedChem Express). The absorbance at 450 nm was measured using an ELx800 plate reader (BioTek Instruments Inc., USA).

### Flow cytometry

The adherent cells were digested using EDTA-trypsin and collected by centrifugation. Then the collected cells were washed with 1 mL of apoptosis detection buffer. APC-Annexin V and of PI (AF647) were added to stain the cells according to the manufacturer’s protocol. Cells were analyzed using the CytoFLEX S flow cytometer (Beckman Coulter Instruments Inc., USA), and the data were analyzed using CytoExpert software (Beckman Coulter Instruments Inc., USA).

### Statistical analysis

All statistical analyses were conducted using the R software (Version 4.3.2; https://www.r-project.org/). Differences between the two groups were analyzed using the Student’s t-test or Wilcoxon test. Prognostic analysis was carried out using the KM method, univariate and multivariate Cox regression analysis. Data visualization was performed using the “ggplot2” R package. In all analyses, *p*-value < 0.05 was considered statistically significant.

## Result

### Four histone deacetylation-related genes were highly associated with prognosis in ccRCC

**Fig. 1:**
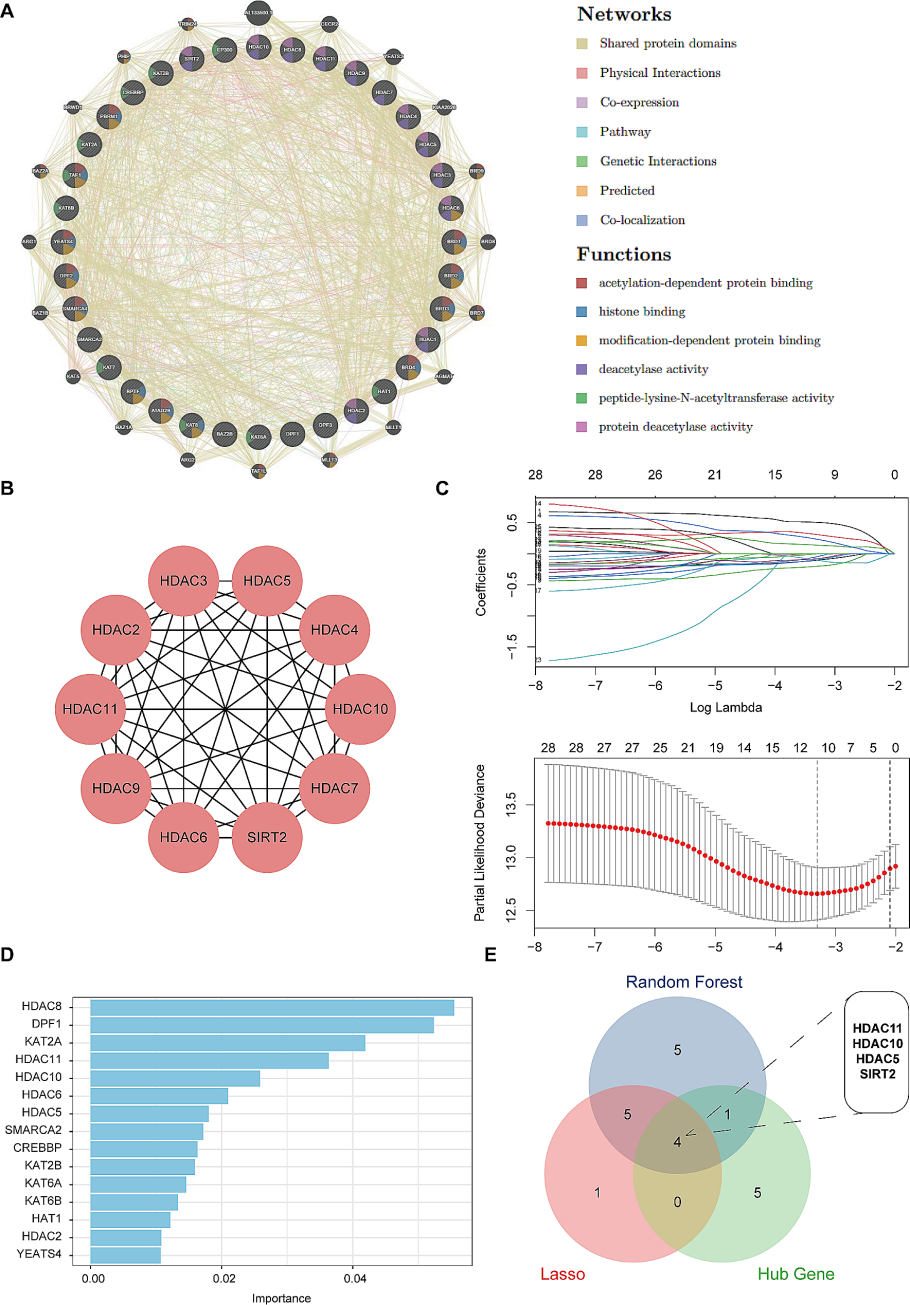
The screening process of the prognostic genes from the 36 acknowledged histone deacetylation genes in ccRCC (**A**) Protein-Protein Interaction Networks based on the 36 histone deacetylation genes. (**B**) The identification of hub genes. (**C**) Coefficient path diagram and Cross-validation of Lasso regression analysis. (**D**) The top 15 genes with variable importance in random forest analysis. (**E**) A Venn diagram showing the intersection of genes. Lasso: least absolute shrinkage and selection operator

The histone deacetylation-related genes were selected from an article published by Yuyan Xu et al., including 9 writers, 12 erasers, and 15 readers (Supplementary Table 1). To further explore the correlation between the above genes, GeneMANIA database was used to construct a PPI network. The results showed that there were extensive interactions between the model genes surrounded by 20 nodes representing genes that were significantly correlated with them. These genes were mainly involved in biological functions, including deacetylation-dependent protein binding, histone binding, modification-dependent protein binding (Fig. 1A). The cytoHubba plugin of Cytoscape (Version 3.10.1) was conducted to screen the hub genes within the PPI network (Fig. 1B). Meanwhile, univariate cox regression analysis was employed to screen the genes with prognostic value preliminarily (Supplementary Table 2). Subsequently, we performed the Lasso and RF analysis to further identify core genes relating with prognosis of KIRC. Lasso regression analysis identified ten candidate genes (Fig. 1C). Additionally, in the Random Forest analysis, the top 15 significant genes were selected for further analysis based on the importance of the variables (Fig. 1D). A Venn diagram showed the overlapping genes of candidate genes from the above three screening analyses, including HDAD11, HDAC10, HDAC5 and SIRT2 (Fig. 1E).

### The aberrant expression and clinical significance of HDAC10 in ccRCC

**Fig. 2:**
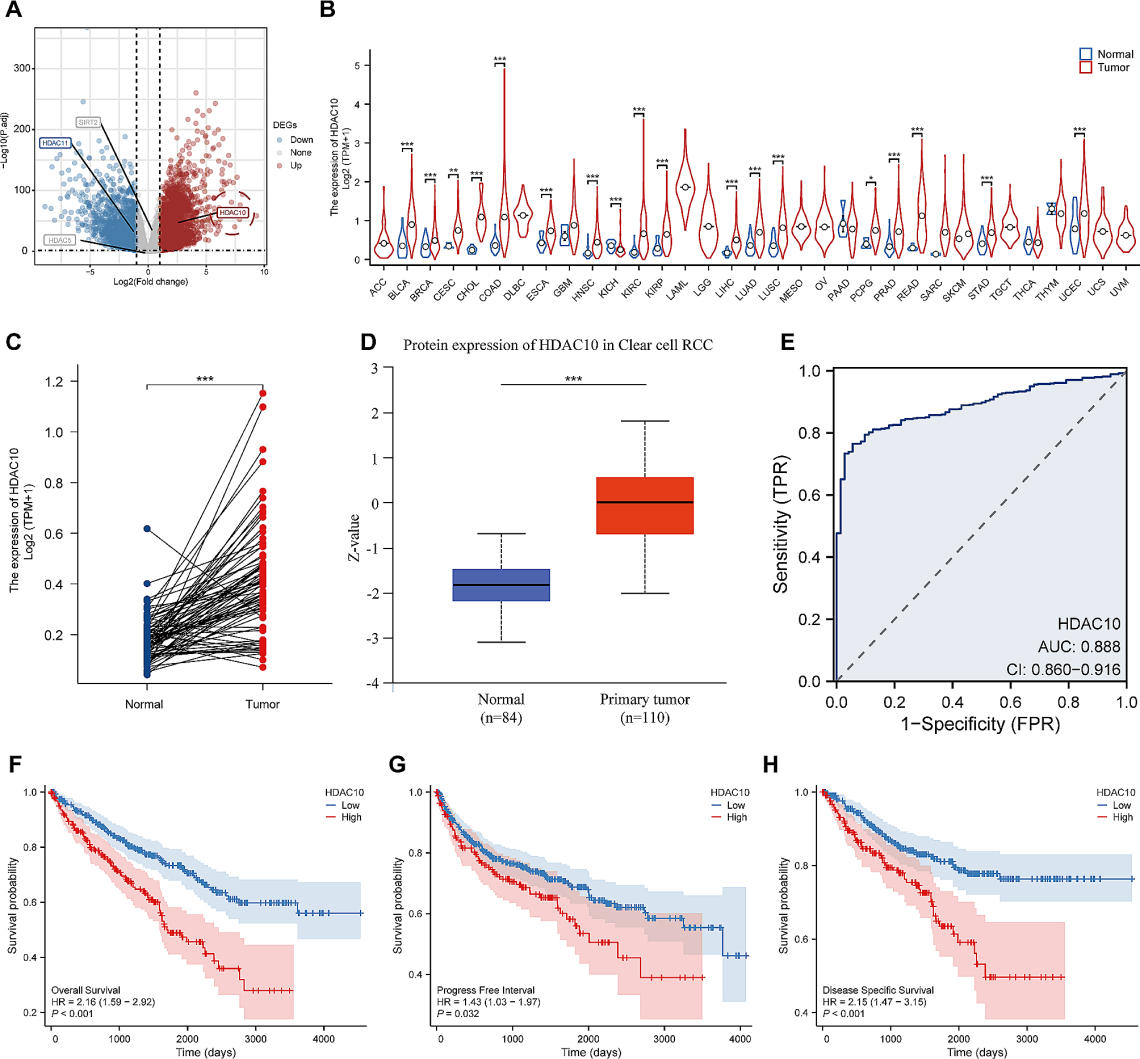
Identification of the differential expression of HDAC10 and in LUAD (**A**) Volcano plot of DEGs between KIRC and normal tissues. (**B**) The expression of HDAC10 in 33 tumor types from the TCGA database. (**C**) The mRNA expression of HDAC10 between paired KIRC and adjacent normal tissues. (**D**) The HDAC10 protein expression between KIRC and normal tissues from the UALCAN database. (**E**) A ROC curve to test the efficiency of HDAC10 to identify KIRC from normal tissue. (F-H) Kaplan-Meier survival curve of OS (**F**), PFI (**G**), and DSS (**H**) between the high and low HDAC10 groups. DEGs: differential expression genes; ROC: receiver operator characteristic; OS: overall survival; DSS: disease specific survival; PFI: progress free interval

We conducted the differential expression analysis between normal and KIRC samples and marked the above four genes with prognostic value. The result showed HDAC10 had the highest fold change level (Fig. 2A). To investigate the expression pattern of HDAC10 in tumors, we conducted a systematic analysis based on TCGA databases. We evaluated the expression of HDAC10 in 33 different types of tumors, which showing that HDAC10 expression varied significantly in 18 different tumors, including Bladder Urothelial Carcinoma (BLCA), Colon adenocarcinoma (COAD), Liver hepatocellular carcinoma (LIHC), Lung adenocarcinoma (LUAD) and KIRC (Fig. 2B). Then, the high expression of HDAC10 in KIRC was further validated by using two GEO datasets (GSE53757 and GSE53000) (Supplementary Fig. 1A-B). In addition, the paired analysis represented HDAC10 was upregulated in KIRC (Fig. 2C). The protein expression data from the UALCAN database also got a similar result (Fig. 2D). The area under the curve (AUC) of HDAC10 is 0.888 in ROC curve analysis, suggesting HDAC10 may be an ideal biomarker to distinguish KIRC from normal tissue (Fig. 2E). Of note, although it has been reported that HDAC10 is expressed differently in various tumors, which was identified in our systematic analysis including 33 different cancer types, its exact role in the initiation and progression of KIRC is not yet clear. In this study, we aim to investigate the underlying mechanism and clinical significance of HDAC10 in KIRC.

After data preprocessing, the relationship between expression of HDAC10 and clinicopathological characteristics of KIRC patients was shown in the baseline data table (Supplementary Table 3). To elucidate the clinical relevance of HDAC10, we performed the prognostic analysis. The KM survival analysis represented that high expression of HDAC10 was associated with poor overall survival (HR = 2.16, 95% CI:1.59–2.92, *p* < 0.001), progress free interval (HR = 1.43, 95% CI: 1.03–1.97, *p* = 0.032) and disease specific survival (HR 2.15, 95% CI: 1.47–3.15, *p* < 0.001) (Fig. 2F-H). The clinical significance of HDAC10 was further validated by the results of KM survival analysis based on four external cohorts (GSE29609, Miao, E-MTAB-1980 and Kaplan-Meier Plotter cohorts) (Supplementary Fig. 1C-F).

### Clinical subgroup analysis and drug sensitivity analysis

**Fig. 3:**
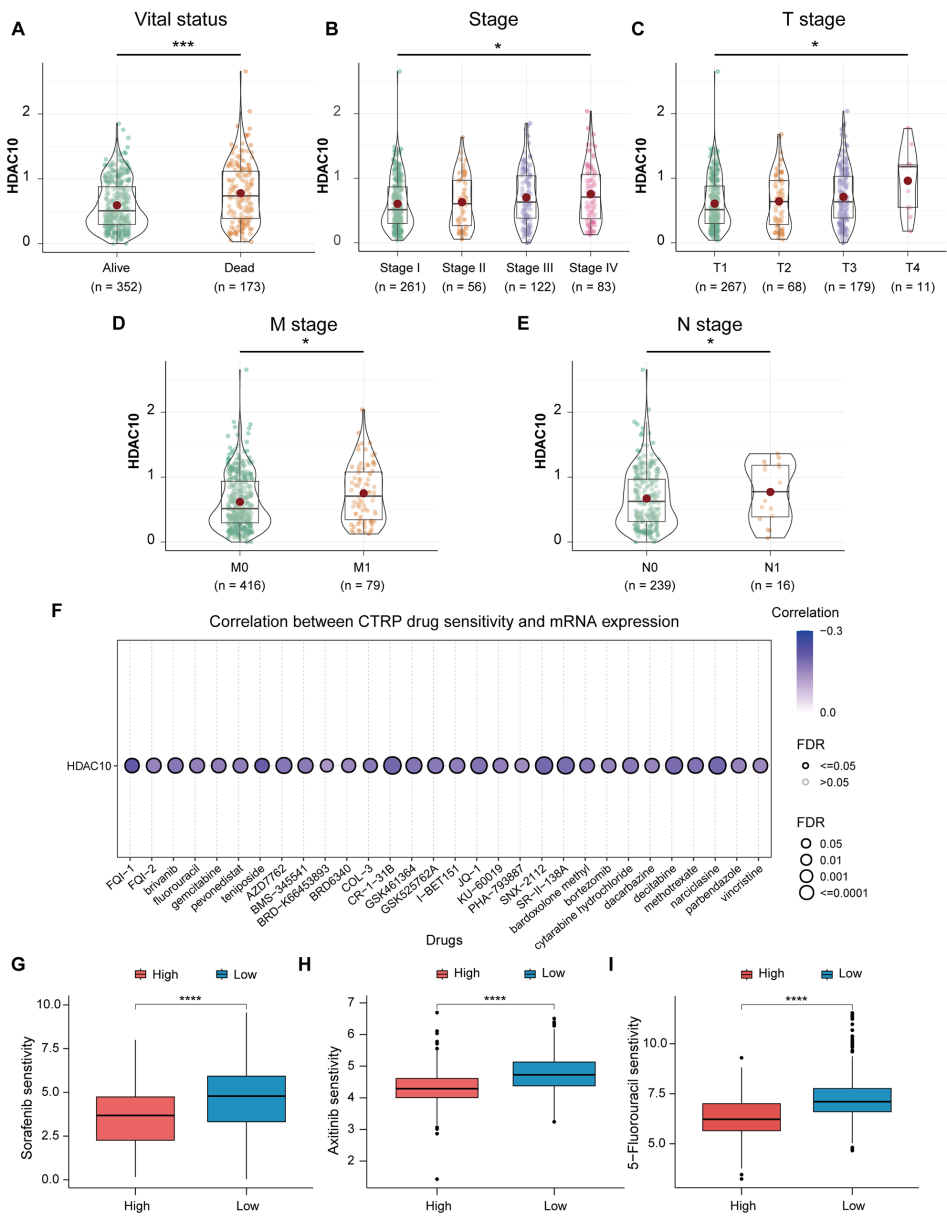
Clinical relevance of HDAC and the drug sensitivity analysis (**A-E**) Clinical subgroup analysis of HDAC10 in survival status (**A**), clinical stage (**B**), T stage (**C**), M stage (**D**), and N stage (**E**). (**F**) The correlation between the expression of HDAC10 and the drug sensitivity from the CTRP database. (**G-I**) Boxplots of IC50 of Sorafenib (**G**), Axitinib (**H**) and 5-Fluorouracil (**I**) in the high and low HDAC10 groups

Subsequently, the result of clinical subgroup analysis revealed that the higher expression of HDAC10 was related to a worse survival outcome, as well as high T, N, and pathologic stages of KIRC (Fig. 3A-D). Nevertheless, no significant difference is observed in the N stage (Fig. 3E). Given that the efficacy of drug therapy, including chemotherapy and targeted drug therapy, is closely related to the prognosis of KIRC, we perform the drug sensitivity analysis subsequently. The relationship between HDAC10 and drug sensitivity was explored using the GSCA database (Fig. 3F). The half-maximal inhibitory concentration (IC50) value for each drug was calculated using the “oncoPredict” package. The results suggested that KIRC patients with high HDAC10 level were sensitive to Sorafenib, Axitinib and 5-Fluorouracil (Fig. 3G-I).

### HDAC10 is upregulated in ccRCC and is associated with poor clinical outcomes

**Fig. 4:**
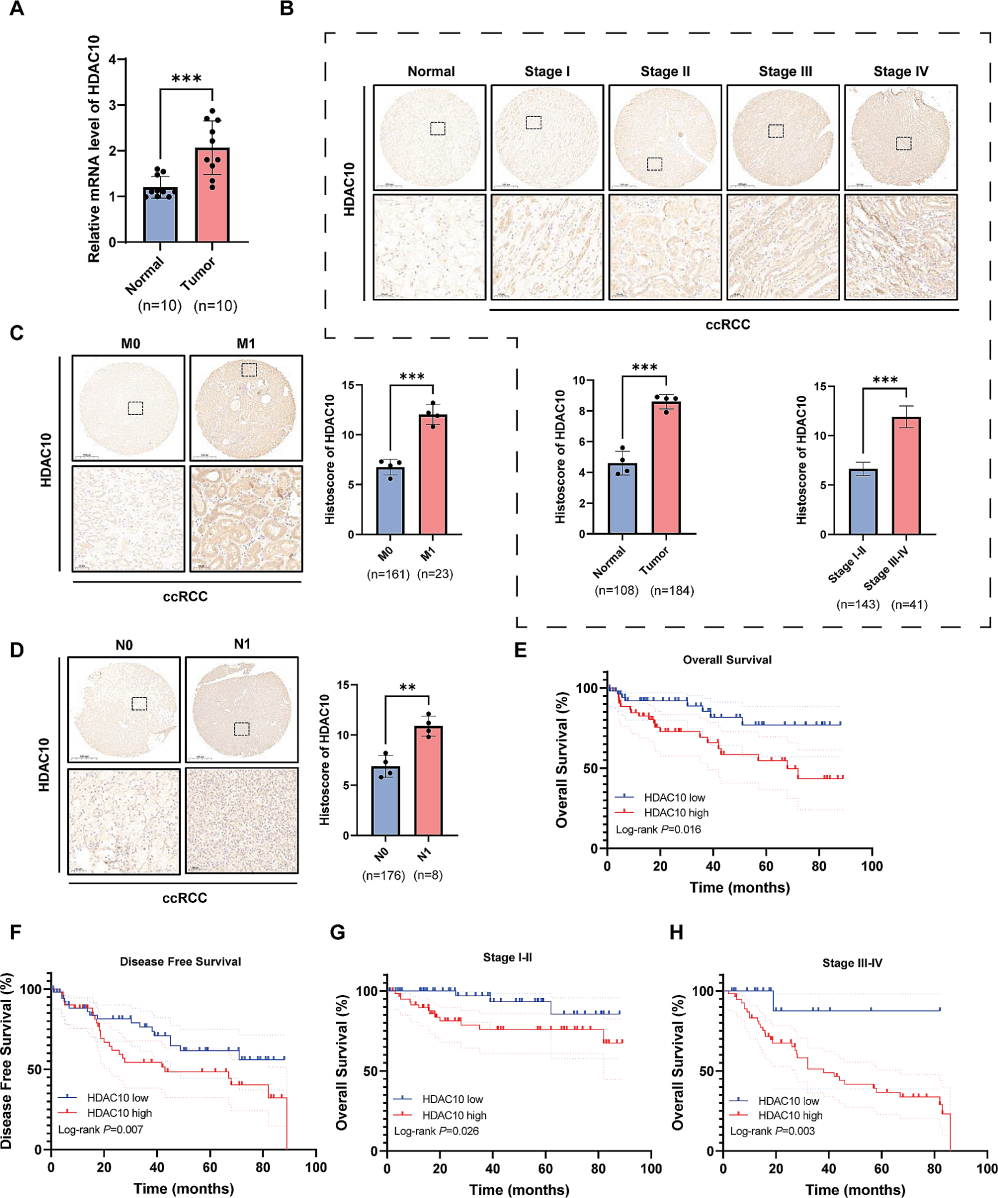
Differential expression validation of HDAC10 and the prognostic analysis in Chinese PLA General hospital cohort **(A)** The expression level of HDAC10 was evaluated by RT-qPCR between the tumor and normal tissues. **(B)** The differential expression of HDAC10 was validated by the IHC between the tumor and normal tissues as well as differential stages. **(C-D)** The IHC was used to compare the expression of HDAC10 at the tissue protein level between the differential M stages **(C)** and N stages **(D)**. **(E-F)** Kaplan-Meier survival curves analysis of OS **(E)** and DFS **(F)** between the high and low HDAC10 groups. **(G-H)** The Kaplan-Meier curves of OS in stage I-II **(G)** and stage III-IV **(H)**. IHC: immunohistochemistry; OS: overall survival; DFS: disease free survival

We collected tumor and paracancerous samples of KIRC from the Chinese PLA General Hospital and obtained the corresponding clinical follow-up data. QRT-PCR experiments were performed to detect the mRNA expression of HDAC10 in ten pairs of KIRC and paracancerous tissues. The results showed that HDAC10 was highly expressed in KIRC tissues (Fig. 4A). In addition, IHC performed on 184 cases of ccRCC tissues and 108 cases of normal renal tissues using tissue microarrays (TMAs) showed that HDAC10 expression was primarily located in the cytoplasm of tumor cells and increased at advanced clinical stages (Fig. 4B). Interestingly, similar results were observed in the N stage and M stage (Fig. 4C-D). In the meantime, KM survival analysis represented that high expression of HDAC10 was associated with poor overall survival (*p* = 0.016) and disease-free survival (*p* = 0.007) (Fig. 4E-F). Further prognostic analysis based on clinical subgroups indicated that compared to patients with low HDAC10 expression, those with stage I-II or III-IV disease and high HDAC10 expression had a poorer prognosis (Fig. 4G-H). Upon comprehensive consideration, both the TCGA data and our clinical data from the Chinese PLA General Hospital cohort indicate that upregulation of HDAC10 lead to malignant progression of ccRCC, potentially serving as a useful prognostic marker for ccRCC.

### The proliferation and invasion of ccRCC cells are significantly affected by the intervention of HDAC10

**Fig. 5:**
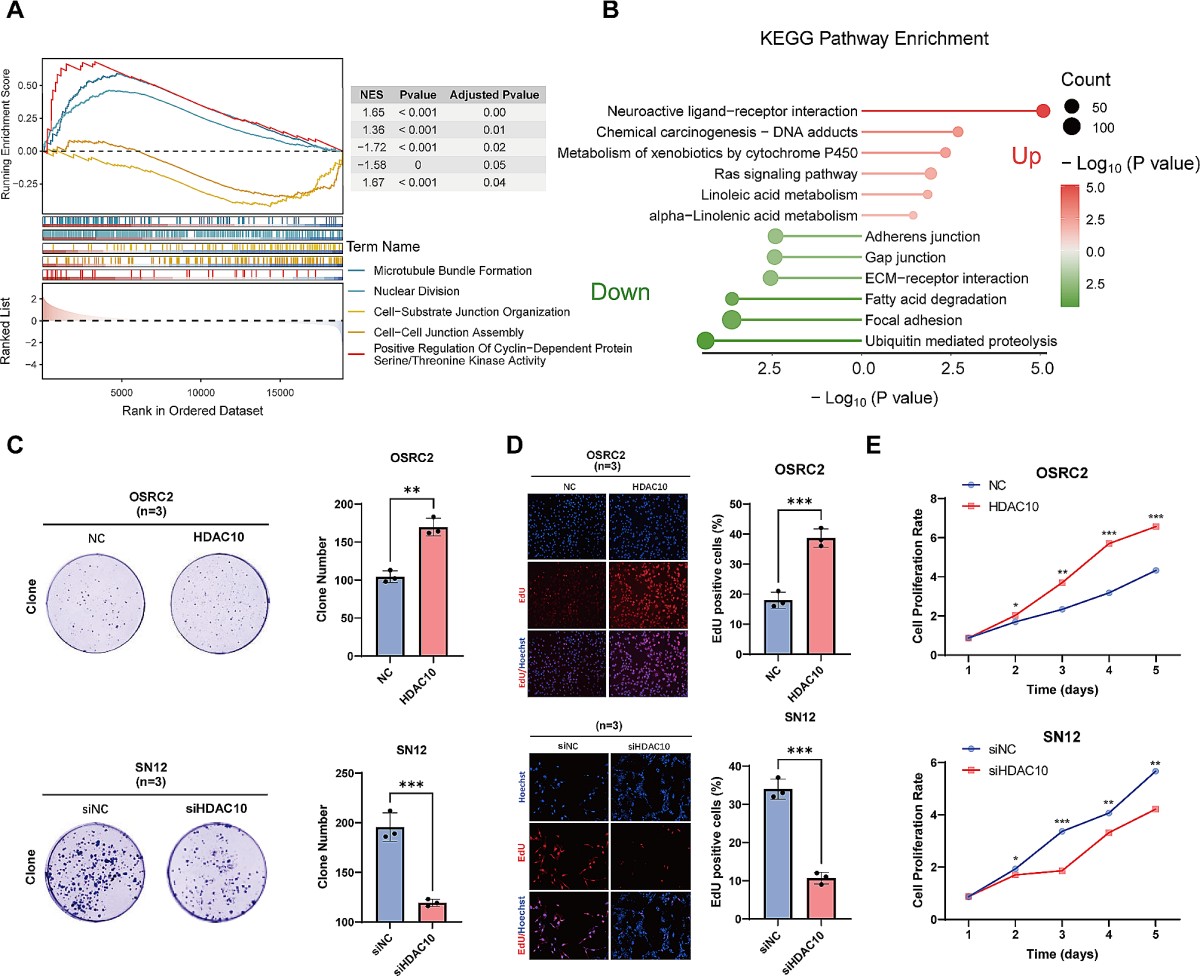
HDAC10 promotes the proliferation of ccRCC cells in vitro. **(A)** GSEA analysis between high and low HDAC10 groups. **(B)** KEGG pathway enrichment analysis between the high and low HDAC10 groups. **(C-E)** The proliferation of ccRCC cells was detected by the colony formation **(C)**, EdU assays **(D)** and CCK-8 **(E)**. GSEA: gene set enrichment analysis; KEGG: Kyoto Encyclopedia of Genes and Genomes

**Fig. 6:**
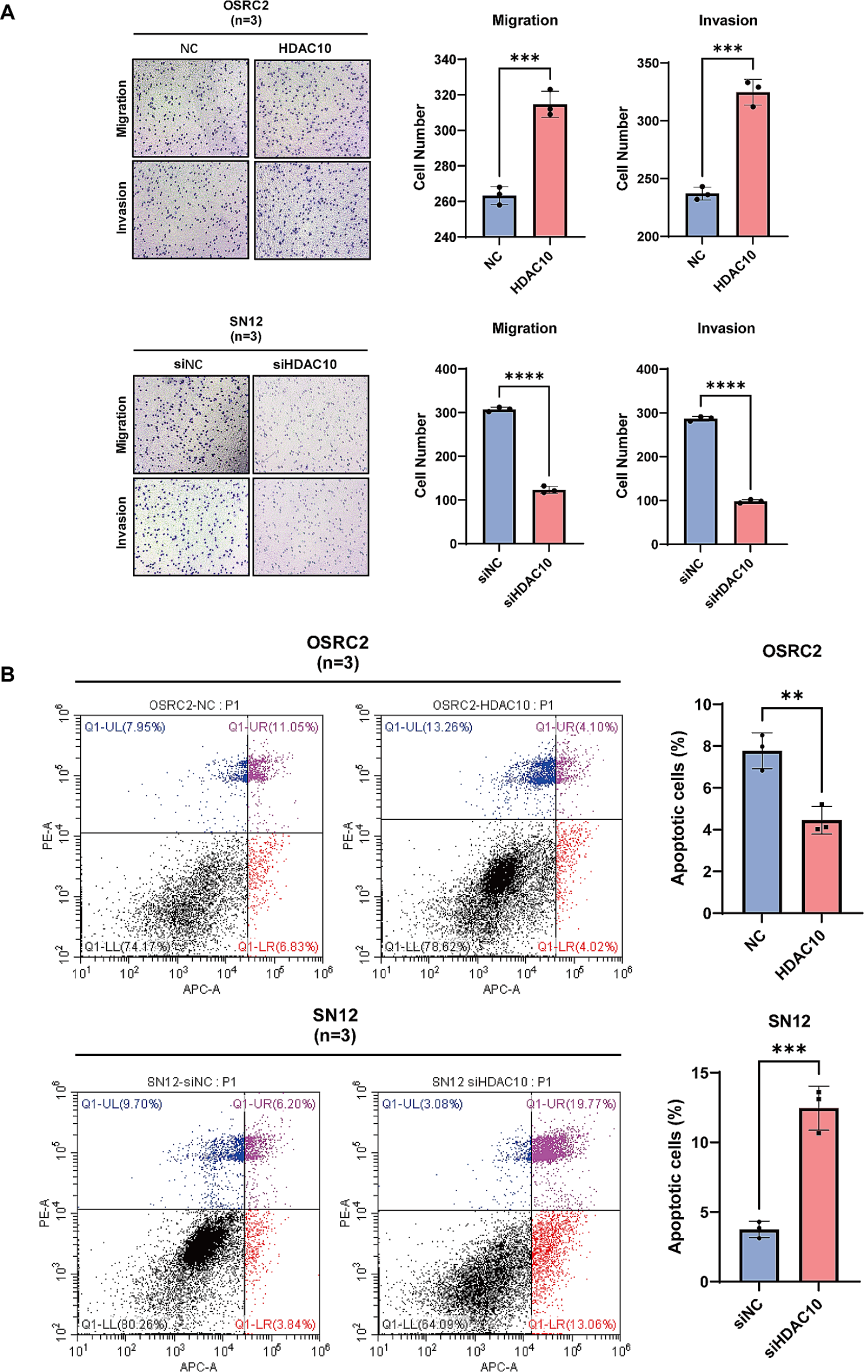
HDAC10 promotes the migration, invasion and apoptosis of ccRCC cells in vitro. **(A)** The migration of ccRCC cells was detected by the transwell migration and invasion assay. **(B)** Flow cytometry analyses and quantitative analyses for apoptosis of OSRC-2 and SN12 cells transduced with control siRNA and siHDAC10 separately

Given that the abnormal expression of HDAC10 has a detrimental effect on the prognosis of KIRC patients, we subsequently attempted to identify the underlying biological mechanisms. Specifically, we identified the DEGs between high-HDAC10 and low-HDAC10 groups based on the median value and performed functional enrichment analysis. Strikingly, GSEA enrichment analysis represented that those DEGs were significantly enriched in microtubule bundle formation, nuclear division, cell − substrate junction organization, cell − cell junction assembly and positive regulation of cyclin − dependent protein serine/threonine kinase activity (Fig. 5A). In addition, KEGG enrichment analysis revealed that pathways related to tumor development were upregulated including chemical carcinogenesis-DNA adducts and Ras signaling pathway. Meanwhile, KEGG pathways of focal adhesion, ECM − receptor interaction, and adherence junction were downregulated significantly (Fig. 5B). The above results implied that HDAC10 mediated the proliferation and metastasis of ccRCC. To elucidate the biological effects of HDAC10 in ccRCC, we initially assessed the expression of HDAC10 in five ccRCC cell lines, including 786-O, OSRC2, A498, SN12-PM6 and Caki-1, through qPCR and western blot experiments (Supplementary Fig. 2A-B). The results indicate that compared to non-metastatic ccRCC cell lines 786-O, OSRC2 and A498, the mRNA and protein expression levels of HDAC10 were significantly elevated in metastatic ccRCC cell lines SN12-PM6 and Caki-1 in consistent. Subsequently, overexpressing-HDAC10 cells (OSRC-2) and knockdown-HDAC10 cells (SN12-PM6) were constructed and observed western blot (Supplementary Fig. 2C-D). We evaluated the impact of HDAC10 expression levels on the proliferation and invasion of ccRCC cells, utilizing EdU assay, CCK-8 assay, cell transwell migration and invasion assay and colony formation assay. The results revealed that in the OE-HDAC10 group, proliferation and invasion capabilities of ccRCC cells were significantly increased. While in KD-HDAC10 group, the proliferation and invasion capabilities of ccRCC cells were significantly decreased (Figs. 5C-E and 6A). The results of Annexin V/PI double staining indicate that the number of apoptotic cells in OE-HDAC10 group was significantly reduced, while the number of apoptotic cells in KD-HDAC10 group was significantly increased. This further suggests that HDAC10 negatively regulates the apoptosis of ccRCC cells, thereby promoting their proliferation. (Fig. 6B)

### The correlation between HDAC10 and the landscape of the immune microenvironment in KIRC

**Fig. 7:**
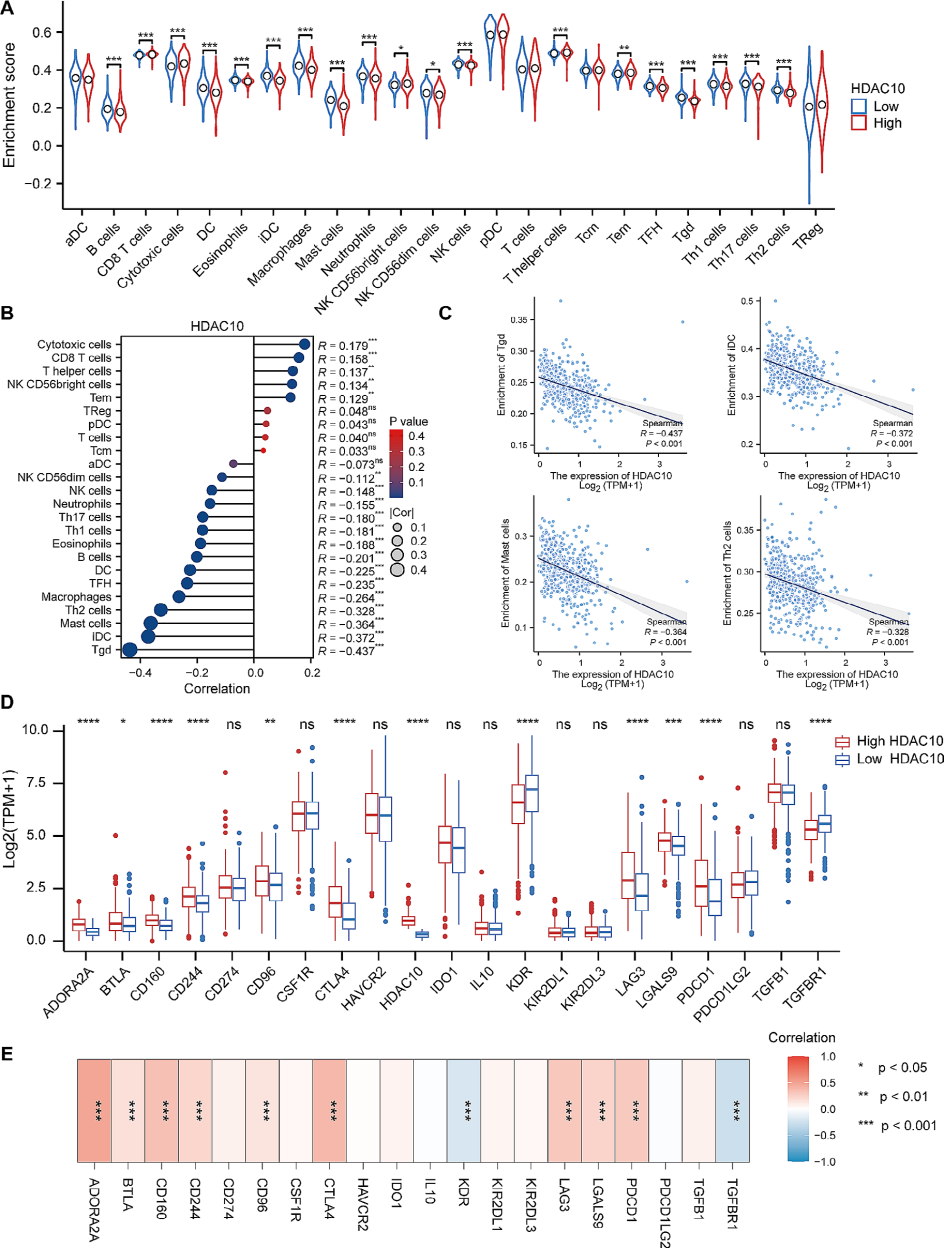
The correlation analysis of immune cell infiltration and immune checkpoints with HDAC10 **(A)** Comparison of the immune infiltration level of 24 immune cell types between high and low HDAC10 groups. **(B)** Correlation between infiltration levels of 24 immune cell types and HDAC10 expression levels by Spearman’s analysis. **(C)** The scatterplots of the top 4 immune cell types with the strongest correlation with HDAC10. (**D**) The differential expression of immune checkpoints between high and low HDAC10 groups. **(E)** The correlation analysis between the immune checkpoints expression level and HDAC10

As reported, infiltration of immune cells significantly impacts tumor development and the prognosis of patients. We performed ssGSEA analysis to quantify the level of infiltration of different immune cells in the tumor microenvironment and found that the level of infiltration of most immune cells was down-regulated in high HDAC10 group compared to the low HDAC10 group among 24 immune cell types, including B cells, dendritic cell (DC), eosinophils cells, immature DC (iDC) cells, macrophages, mast cells, T gamma delta (Tgd) cells, Th1 cells, Th17 cells, Th2 cells (Fig. 7A). Consistently, further Spearman correlation analysis showed that the expression level of HDAC10 was negatively correlated with most immune cell types, such as Tgd cells, iDC, mast cells and Th2 cells (Fig. 7B-C).

Tumor cells can evade immunosurveillance and progress in various ways, one of which is through the activation of immune checkpoint pathways that cause T-cell exhaustion and suppress antitumor immune responses. So, we wanted to investigate if there was a correlation between immune checkpoints from the TISIDB database in the high and low HDAC10 groups. Our result represented that the level of multiple immune checkpoints such as LAG3, PDCD1 and CTLA4 was up-regulated in the high HDAC10 group (Fig. 7D). Additionally, our correlation analysis showed that most immune checkpoints genes showed significantly positive correlation with HDAC10, including ADORA2A, CD160, PDCD1 and CTLA4 (Fig. 7E), which implied that patients in the high HDAC10 group have impaired immune function and their unfavorable prognosis may at least partly relate to T-cell exhaustion.

### Establishment and assessment of the prognostic nomogram model

**Fig. 8:**
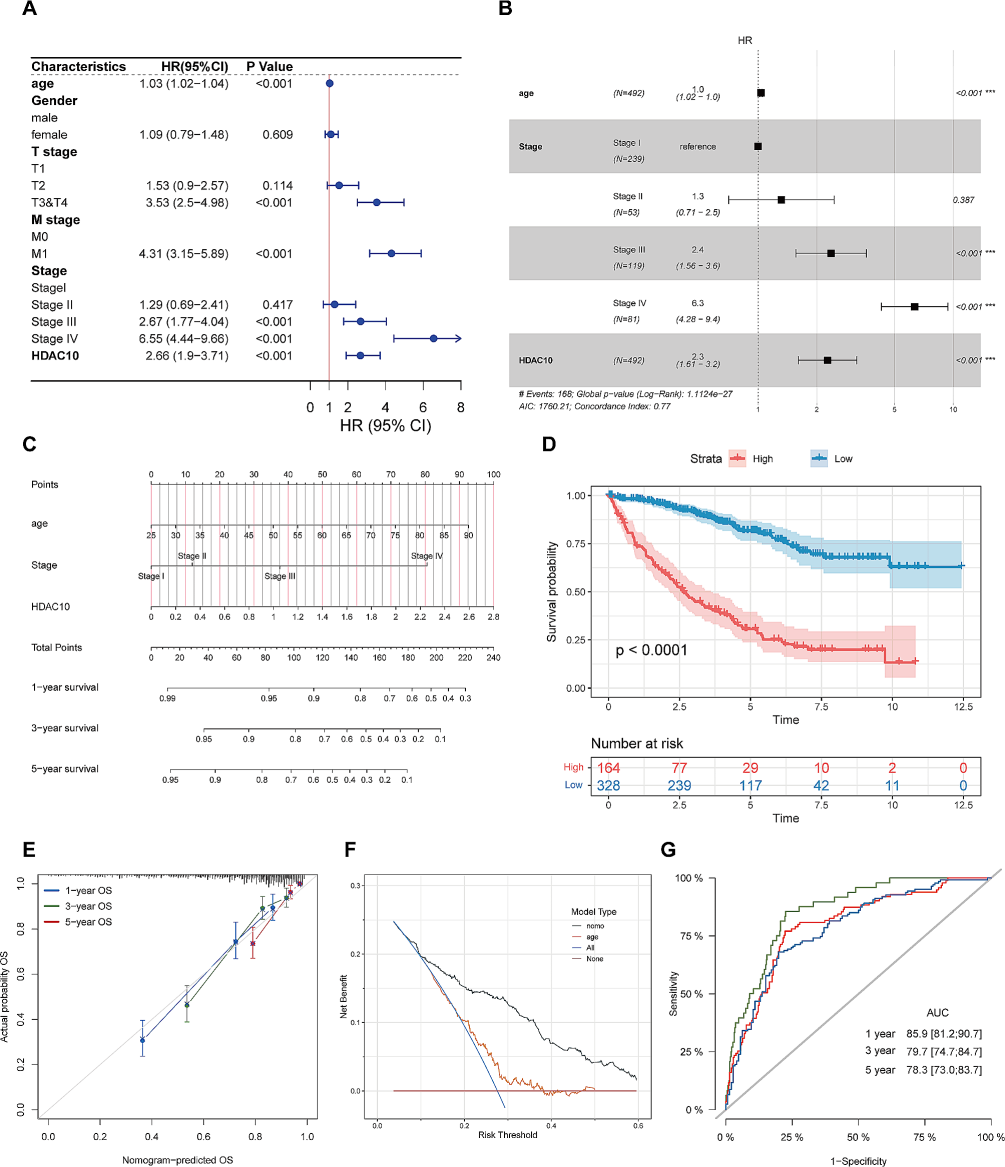
Construction and assessment of the nomogram survival model **(A)** Univariate analysis of HDAC10 and the clinicopathologic characteristics. **(B)** Multivariate analysis of HDAC10 and the clinicopathologic characteristics. **(C)** A nomogram integrating HDAC10 and clinicopathologic characteristics. **(D)** A survival curve between the high and low nomogram score groups. **(E)** The calibration curve of the nomogram. **(F)** Decision curve analysis (DCA) of the nomogram. **(G)** Receiver operator characteristic (ROC) analysis of the nomogram

To provide a quantitative approach to predict prognosis of KIRC, we attempted to incorporate HDAC10 with clinicopathological characteristics for establishing a clinical prognostic model. We performed the univariate and multivariate cox regression analyses. The result of the univariate analysis revealed that HDAC10 had a significant association with OS (HR: 2.66; 95% CI:1.9–3.71; *p* < 0.001), as well as with T, M and pathological stage (Fig. 8A). After adjusting for other confounding factors, the multivariate analysis also indicated that HDAC10 was an independent prognostic risk factor (HR:2.3; 95% CI:1.61–3.2; *p* < 0.001) (Fig. 8B). Multivariable Cox and stepwise regression analyses were employed to construct the optimal nomogram model in the TCGA-KIRC cohort with the minimum AIC (Akaike information criterion). Age, pathological stage, and HDAC10 were included in this mode (Fig. 8C). A significant survival difference was found between the high- and low nomogram score group (Fig. 8D). The calibration curve of the nomogram showed that its 1-, 3-, and 5-year survival rates closely matched the ideal line (the 45-degree line), reflecting the high accuracy of our model (Fig. 8E). The result of the decision curve analysis (DCA) indicated that the nomogram could obtain a considerable net benefit (Fig. 8F). The ROC curve demonstrated the reliability of the nomogram model in predicting patient survival at 1-, 3-, and 5-year intervals, indicating the accuracy of this model in predicting OS of CRC patients (AUC: 1-year: 0.859, 95% CI: 0.812–0.907; 3-year: 0.797, 95% CI: 0.747–0.847 1; 5-year: 0.783, 95% CI: 0.730–0.837) (Fig. 8G). A user-friendly website has been established to make it easier for clinicians to utilize our prediction model ( https://nomogramhdac10.shinyapps.io/HDAC10_Nomogram/ ). The website will automatically generate a survival plot and provide predicted survival time for patient with input of related data.

## Discussion

ccRCC is the most common type of RCC, causing over 175,000 deaths per year globally. Of note, it has been reported that 30–35% of patients showed distant metastasis after surgery [1]. Despite advances in the fields of surgery, chemotherapy, radiotherapy, target therapy, and immunotherapy, the prognosis of ccRCC patients, especially those in advanced stages, remains very gloomy due to high heterogeneity of ccRCC [4, 32, 33]. Extensive heterogeneity is an essential characteristic of tumors, which may result in diverse patient clinical outcomes. Although many efforts have been dedicated toward elucidating tumor heterogeneity, our knowledge is still limited.

Acetylation, as the first discovered modification, is the most studied and best characterized among all modifications [34]. There is compelling evidence that deacetylation of lysine residues at amino acid termini greatly affects transcriptional regulations. HDAC serves as an ‘eraser’, which remove acetate from acetylated histone as well as other non-histone proteins [7]. Extensive research has revealed a strong correlation between histone deacetylation and tumorigenesis, significantly influencing critical biological functions in tumor cells such as proliferation, apoptosis, and metastasis [35–37]. HDACs play vital role in regulating gene expression by removing acetyl groups from histones. A multitude of research has consistently shown irregular expression patterns of HDACs (HDAC1, HDAC5, and HDAC7) in various human tumors(38. HDAC inhibitors (HDACi) can trigger hyperacetylation of histones, contributing to reactivation of tumor-suppressor genes for impeding tumor growth. Many HDACi have demonstrated effective anti-tumor properties in several hematological and solid malignancies [39, 40].

In our study, based on two machine learning algorithms and PPI network analysis, we identified HDAC10 as a key gene related to the prognosis of ccRCC. HDAC10, as a member of the class II HDACs, was reported by Fischer et al., Tong et al. and Kao et al. in 2002 [41, 42]. Recent studies have shown that HDAC10 plays a role in the occurrence and development of various tumors. For instance, it has been reported that HDAC10 can combine with Pax3 and KAP1 to form a ternary complex and inhibit their expression and activity to promote melanogenesis, and only HDAC10 expression levels significantly correlate with poor overall survival in neuroblastoma patients among the 11 metal-dependent HDACs [43]. In addition, Cheng et al. implied that HDAC8, HDAC10, and HDAC11 may serve as potential molecular biomarkers and therapeutic targets for ccRCC [44]. Similarly, we found that HDAC10 was highly expressed in ccRCC at transcriptome and proteome levels, and prognostic analysis demonstrated its high expression was associated with poor prognosis of ccRCC patients. The aberrant expression of HDAC10 in ccRCC was validated by PCR and IHC in the ccRCC patients from the Chinese PLA General Hospital. Further univariate and multivariate analysis revealed that HDAC10 could be considered an independent prognostic risk factor in ccRCC.

Given the critical clinical significance of HDAC10 in ccRCC but its biological function of HDAC10 affecting the carcinogenesis and progression of ccRCC remained unclear, we subsequently performed GSEA and KEGG functional analysis. The results demonstrated that multiple carcinogenesis-related pathways, including Ras signaling pathway and chemical carcinogenesis-DNA adducts, were activated. However, KEGG pathways of focal adhesion, ECM − receptor interaction, and adherence junction as well as the GO terms of cell − substrate junction organization and cell − cell junction assembly were downregulated significantly. The above results suggest that HDAC10 may mediate the proliferation and metastasis of tumor cells. The following colony-formation assay, EdU, and CCK8 experiments showed that overexpression of HDAC10 can promote the proliferation of tumor cells while knockdown of HDAC10 can significantly inhibit the process. In addition, the result of cell transwell migration and invasion assay demonstrated the promotional effect of HDAC10 on cell migration and invasion.

Accumulating evidence suggests the tumor microenvironment (TME), especially tumor-infiltrating immune cells (TIICs), plays a critical role in the tumorigenesis and progression of KIRC [45]. In recent years, numerous cancer immunotherapy strategies, represented by the immune checkpoint inhibitors (ICIs) have had a revolutionary influence on the treatment of KIRC 46). However, only a small percentage of patients achieved a durable immune response after treatment [47]. The underlying mechanisms are far from being elucidated. Interestingly, researchers have found that histone deacetylation is closely related to TME [48–50]. In our study, we found the expression level of HDAC10 was significantly negatively correlated with most immune cell types, including Tgd cells, iDC and NK cells, which implied HDAC10 may influence the tumor antigen expression, antigen process and impair the function of the innate immunity system. In addition, it is noteworthy that the infiltration level of CD8 + T cell was higher in the high HDAC10 group than in the low HDAC10 group while the expression level of HDAC10 was positively correlated with the level of CD8 + T-cell infiltration. However, the following analysis revealed that HDAC10 was significantly positively correlated with the expression levels of various immune checkpoints. Specifically, the levels of multiple immune checkpoints were up-regulated in the high HDAC10 group, including the key immunotherapeutic markers PDCD1 and CLAT4. As reported, exhausted T cells lose effective immune functions, leading to the immune escape of cancer cells. It is well recognized that the expression of immune checkpoints contributes greatly to this exhausted phenotype of T cells and affects the prognosis of patients [51]. This implied that patients in the high HDAC10 group have impaired immune function and their unfavorable prognosis may at least partly relate to the T-cell exhaustion.

In summary, based on two different machine learning algorithms and PPI network analysis, we have identified HDAC10 as a crucial regulator for the prognosis of KIRC. Our bioinformatics analysis and the results of PCR and IHC revealed that HDAC10 was highly expressed in KIRC and associated with poor prognosis. Additionally, HDAC mediates tumor cell proliferation, invasion, and metastasis, as well as affect the TME homeostasis in ccRCC. HDAC10 is a pro-oncogenic regulator and could be a potential target for the treatment of ccRCC. Nevertheless, the patients included in the study were mainly obtained from open databases and recruited retrospectively, which may inevitably lead to bias. Moreover, the underlying mechanisms of HDAC10 for affecting the tumor cells and the TME need to further elucidated at the cellular and molecular levels.

## Electronic supplementary material

Below is the link to the electronic supplementary material.

Supplementary Material 1

Supplementary Material 2

Supplementary Material 3

Supplementary Material 4

Supplementary Material 5

## Data Availability

No datasets were generated or analysed during the current study.
