# Supplementary figures and images for: Identification of HDAC10 as a candidate oncogene in clear cell renal carcinoma that facilitates tumor proliferation and metastasis
Source: Diagn Pathol. 2024 Sep 5;19:120. doi: 10.1186/s13000-024-01493-2 (PMC11378624; doi:10.1186/s13000-024-01493-2)

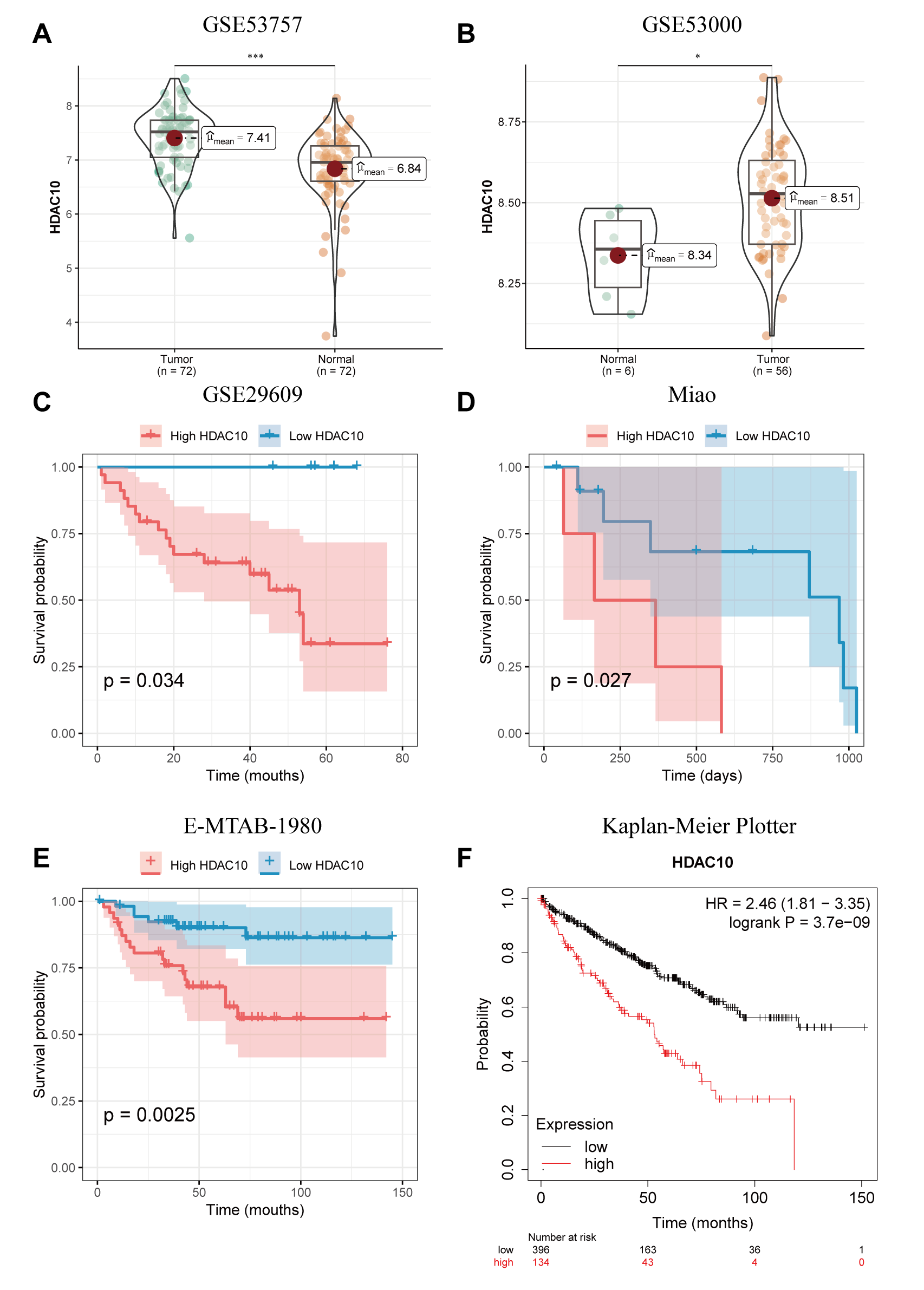

Supplement: Supplementary file 1 — Supplementary Material 1 [file 13000_2024_1493_MOESM1_ESM.tif]

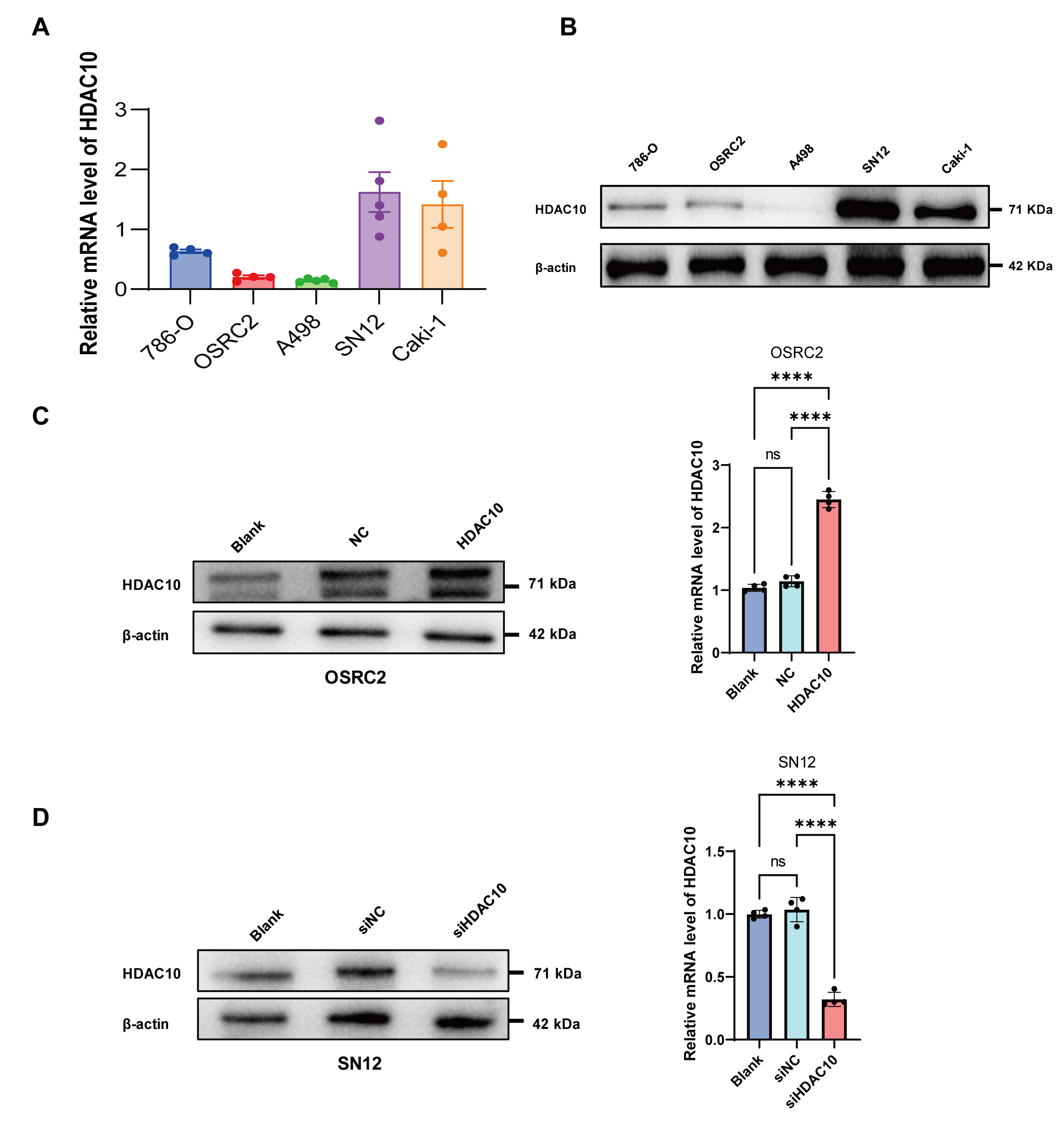

Supplement: Supplementary file 2 — Supplementary Material 2 [file 13000_2024_1493_MOESM2_ESM.tif]
