# Supplementary material for: Identification of HDAC10 as a candidate oncogene in clear cell renal carcinoma that facilitates tumor proliferation and metastasis
Source: Diagn Pathol. 2024 Sep 5;19:120. doi: 10.1186/s13000-024-01493-2 (PMC11378624; doi:10.1186/s13000-024-01493-2)
Supplement: Supplementary file 5 — Supplementary Material 5 [file 13000_2024_1493_MOESM5_ESM.docx]

| **Characteristics** | **Low expression of HDAC10** | **High expression of HDAC10** | **P value** |
| --- | --- | --- | --- |
| n | 270 | 271 |  |
| Pathologic T stage, n (%) |  |  | 0.028 |
| T1 | 152 (28.1%) | 127 (23.5%) |  |
| T2&T4&T3 | 118 (21.8%) | 144 (26.6%) |  |
| Pathologic N stage, n (%) |  |  | 0.844 |
| N0 | 112 (43.4%) | 130 (50.4%) |  |
| N1 | 7 (2.7%) | 9 (3.5%) |  |
| Pathologic M stage, n (%) |  |  | 0.040 |
| M0 | 233 (45.9%) | 196 (38.6%) |  |
| M1 | 33 (6.5%) | 46 (9.1%) |  |
| Pathologic stage, n (%) |  |  | 0.021 |
| Stage I&Stage II | 179 (33.3%) | 153 (28.4%) |  |
| Stage III&Stage IV | 90 (16.7%) | 116 (21.6%) |  |
| Primary therapy outcome, n (%) |  |  | 0.866 |
| CR&PR | 64 (43.5%) | 66 (44.9%) |  |
| PD&SD | 8 (5.4%) | 9 (6.1%) |  |
| Gender, n (%) |  |  | 0.092 |
| Female | 84 (15.5%) | 103 (19%) |  |
| Male | 186 (34.4%) | 168 (31.1%) |  |
| Age, n (%) |  |  | 0.465 |
| <= 60 | 130 (24%) | 139 (25.7%) |  |
| > 60 | 140 (25.9%) | 132 (24.4%) |  |
| Race, n (%) |  |  | < 0.001 |
| Asian | 1 (0.2%) | 7 (1.3%) |  |
| Black or African American | 17 (3.2%) | 40 (7.5%) |  |
| White | 248 (46.4%) | 221 (41.4%) |  |
| Histologic grade, n (%) |  |  | 0.284 |
| G1&G2 | 130 (24.4%) | 120 (22.5%) |  |
| G3&G4 | 134 (25.1%) | 149 (28%) |  |
| Serum calcium, n (%) |  |  | 0.209 |
| Low | 111 (30.2%) | 93 (25.3%) |  |
| Normal | 74 (20.2%) | 79 (21.5%) |  |
| Elevated | 3 (0.8%) | 7 (1.9%) |  |
| Hemoglobin, n (%) |  |  | 0.410 |
| Low | 131 (28.4%) | 133 (28.9%) |  |
| Normal | 107 (23.2%) | 85 (18.4%) |  |
| Elevated | 3 (0.7%) | 2 (0.4%) |  |
| Laterality, n (%) |  |  | 0.101 |
| Left | 117 (21.7%) | 136 (25.2%) |  |
| Right | 153 (28.3%) | 134 (24.8%) |  |
